# Calcific Aortic Valve Disease Is Associated with Layer-Specific Alterations in Collagen Architecture

**DOI:** 10.1371/journal.pone.0163858

**Published:** 2016-09-29

**Authors:** Heather N. Hutson, Taylor Marohl, Matthew Anderson, Kevin Eliceiri, Paul Campagnola, Kristyn S. Masters

**Affiliations:** 1 Department of Biomedical Engineering, University of Wisconsin-Madison, Madison, Wisconsin, United States of America; 2 Laboratory for Optical and Computational Instrumentation, University of Wisconsin-Madison, Madison, Wisconsin, United States of America; University of California, San Diego, UNITED STATES

## Abstract

Disorganization of the valve extracellular matrix (ECM) is a hallmark of calcific aortic valve disease (CAVD). However, while microarchitectural features of the ECM can strongly influence the biological and mechanical behavior of tissues, little is known about the ECM microarchitecture in CAVD. In this work, we apply advanced imaging techniques to quantify spatially heterogeneous changes in collagen microarchitecture in CAVD. Human aortic valves were obtained from individuals between 50 and 75 years old with no evidence of valvular disease (healthy) and individuals who underwent valve replacement surgery due to severe stenosis (diseased). Second Harmonic Generation microscopy and subsequent image quantification revealed layer-specific changes in fiber characteristics in healthy and diseased valves. Specifically, the majority of collagen fiber changes in CAVD were found to occur in the spongiosa, where collagen fiber number increased by over 2-fold, and fiber width and density also significantly increased. Relatively few fibrillar changes occurred in the fibrosa in CAVD, where fibers became significantly shorter, but did not otherwise change in terms of number, width, density, or alignment. Immunohistochemical staining for lysyl oxidase showed localized increased expression in the diseased fibrosa. These findings reveal a more complex picture of valvular collagen enrichment and arrangement in CAVD than has previously been described using traditional analysis methods. Changes in fiber architecture may play a role in regulating the pathobiological events and mechanical properties of valves during CAVD. Additionally, characterization of the ECM microarchitecture can inform the design of fibrous scaffolds for heart valve tissue engineering.

## Introduction

The aortic heart valve is comprised of three distinct extracellular matrix (ECM) layers: the fibrosa, spongiosa, and ventricularis. Type I collagen is the predominant component of the fibrosa in healthy valves, while the spongiosa and ventricularis are primarily composed of proteoglycans and a collagen/elastin network, respectively [1]. This trilaminar structure is believed to be important in guiding both the biological and mechanical functions of the aortic valve [2], and its disruption occurs early in the development of calcific aortic valve disease (CAVD). Although valve failure is typically associated with extensive calcification of the valve structure, even mild leaflet fibrosis and thickening in the absence of calcification is correlated with an increase in cardiovascular and all-cause mortality [3, 4]. These consequences of leaflet thickening highlight the critical role of the ECM in maintaining valvular function, but the precise role and timing of altered ECM remodeling within the cascade of CAVD events remain unknown [5, 6].

Several studies in recent years have illustrated the powerful influence of the valvular ECM in regulating pathological events in valve tissues or cultures. For instance, 2-D investigations of valvular interstitial cells (VICs) cultured on different ECM coatings demonstrated that ECM identity can modulate VIC differentiation to a myofibroblastic or osteoblastic phenotype [7–9]. Forced disruption of targeted ECM components via enzymatic treatment of native leaflets provided 3-D evidence that confirmed the aforementioned 2-D findings [8, 10]. Examination of aortic valve leaflets from swine with homozygous familial hypercholesterolemia, a disease associated with a 1-in-2 chance of CAVD in humans [11, 12], revealed that ECM disorganization and the consequent leaflet thickening preceded almost all other hallmarks typically associated with CAVD [13].

The majority of valve ECM investigations have focused on bulk changes in ECM composition, but several studies have also examined nano- and micro-scale architecture in the valves. These examinations of ECM architecture in healthy valves from humans [14] and animals [15–19] have yielded valuable insight into fiber-level contributions to the unique mechanical properties of the valve. In highlighting the importance of fiber architecture in regulating the valvular response to mechanical loading, these studies also provide strong motivation to pursue quantification of ECM microarchitecture in human valves with CAVD, where altered valve mechanical performance plays a significant role in valve failure. The microarchitecture of ECM components can not only contribute to tissue mechanics, but also provide contact guidance cues that are highly influential in regulating cell function. Fibril/fiber features in type I collagen, such as length, thickness, alignment, and density can regulate cellular behaviors such as cell polarity, motility, proliferation, and differentiation [20]. Culture of various cell types on substrates patterned with nano/micro-topographical features, as well as within scaffolds with varied fiber characteristics, have revealed numerous, highly specific architecture-dependent cellular phenomena, including downregulated fibrotic activity and promotion of a quiescent phenotype in corneal fibroblasts cultured on aligned fibers [21], increased motility of fibroblasts in areas of pattern anisotropy [22], or leading edge localization of focal adhesions and greater cytoskeletal alignment on dense, but not sparse, nanoscale grooves [23].

In the present study, we determine whether CAVD is associated with microarchitectural remodeling by quantifying collagen fiber characteristics, distribution, and crosslinking enzymes in age-matched healthy and diseased human aortic valves. Because standard histological and microscopy techniques can provide only limited information about ECM architecture, Second Harmonic Generation (SHG) microscopy was employed to accomplish this goal. SHG is an imaging technique that allows for direct imaging of collagen architecture without the need for exogenous stains or dyes [24]. The characterization of collagen structures enabled by this analysis can provide further insight into the pathological events that are capable of regulating both the biological and mechanical behaviors of valves during CAVD.

## Methods and Materials

### Reagents and Materials

All reagents used were purchased from Sigma Aldrich (St. Louis, MO) unless otherwise indicated.

### Tissue Acquisition and Classification

Aortic valve leaflets from individuals 50–75 years of age were collected from the University of Wisconsin Hospital and the William S. Middleton Veterans Memorial Hospital in Madison, WI. Healthy aortic heart valves were obtained within 24 hours post-mortem from individuals with no previous diagnosis of CAVD; this protocol was reviewed by the UW-Madison IRB and granted an exemption (#2012–0721) due to not qualifying as human subjects research as defined under 45 CFR 46.102(f). Diseased valves were obtained from individuals undergoing aortic valve replacement surgery due to CAVD. Written consent was obtained from patients by the cardiothoracic surgery team, in accordance with protocol #2009–1094 approved by the UW-Madison IRB. These valves were confirmed as diseased by the cardiothoracic surgery team and a pathologist, and all had macroscopically visible and palpable calcification (example pictured in S1 Fig). Tissues were embedded in paraffin and sectioned in 5 μm slices for histological staining or 13–15 μm slices for SHG imaging. Classification of valves as healthy or diseased was also confirmed via histological analysis. Specifically, the width of leaflet cross-sections that had undergone Movat’s pentachrome staining was measured using ImageJ software [25] at 40 different locations within the tissue interior (i.e., a minimum of 100 μm from the free and attached edges). Leaflets < 700 μm thick were considered healthy.

### Histological Staining

Deparaffinized tissue sections were stained with Movat’s pentachrome for connective tissue (Poly Scientific R&D Corp., Bay Shore, NY), mounted using Permount, and imaged using an Olympus IX51 inverted microscope. Picrosirius red staining was conducted as previously described [26] on sections from the belly region of the leaflet. Briefly, following deparaffinization tissue sections were stained using 0.1% (w/v) Direct Red 80 in saturated aqueous picric acid solution for one hour then washed twice in acidified water, followed by dehydration in graded washes of ethanol and xylene. Sections were mounted using Permount and imaged on an Olympus BX60 upright microscope with a DP25 camera using CellSens Standard software (V1.13) for both bright field and linearly polarized birefringence. Using FIJI (open source, version 2.0.0-rc-43/1.5e) [27] the birefringence of three areas of equal dimensions per tissue layer per image was measured. The birefringence of each region of interest was quantified using a method previously described [28].

### Second Harmonic Generation Imaging

In preparation for SHG imaging, tissue sections of the belly region of the leaflet were deparaffinized in xylene, followed by graded washes in ethanol before being mounted with coverslips sealed with lacquer. Prepared sections were stored at 4°C and imaged within 2 weeks. SHG imaging of collagen was achieved using a mode-locked 890 nm laser. SHG was measured at 445 nm with a 20 nm bandpass filter. As described elsewhere [29], SHG images were acquired using a 40x water-immersion objective, (working distance 3mm, 0.8 NA) on an Olympus BX61 upright microscope (Olympus, Tokyo, Japan) using Olympus Fluoview 300 scanning system at a 1x digital zoom.

### Fiber Analysis

To quantify fiber characteristics, three SHG z-stacks were taken per tissue section from five diseased specimens and five healthy specimens of similar age. Images within the z-stack were taken every 1 μm throughout the entire section. Three serial images from the interior of each stack were analyzed together to negate signal changes due to tissue edge effects and to better characterize fibers angled out of a single plane. Analysis of fiber structure was performed using ctFIRE V1.3 Beta [30] and CurveAlign V3.0 Beta2, both of which are open source software developed by the Laboratory for Optical and Computational Instrumentation (LOCI) at the University of Wisconsin-Madison. SHG images (in 8-bit tiff format) were imported into the program where they underwent curvelet transform (CT) reconstruction through use of CurveLab 2.1.2 (April 2008) followed by creation of fiber overlays via the FIRE algorithm [31]. Measurements from ctFIRE then underwent feature analysis through CurveAlign enabling output of final fiber features. MATLAB (The MathWorks, Inc., Natick, MA) was used as the platform to run the ctFIRE and CurveAlign software. The data from selected fibers were then exported from CurveAlign in Excel file format (Microsoft, Redmond, WA) to undergo statistical analysis.

The analysis described above yielded quantification of the following fiber features: length, width, curvature, density, and alignment. Fiber total length refers to the full length of the fiber within the plane of view, tracing along any curving or crimping the fibers may display, while the end-to-end length measurement is the straight distance from one end of the fiber to the other end, without tracing along the fiber. Curvature is a ratio of these two characteristics, thereby describing the degree of curving and/or crimping the fibers display. Fiber width is the average width of the fiber along its length. Fiber density and alignment were measured in relation to the nearest 2, 4, 8, and 16 nearest neighboring fibers as well as in image regions of defined area: 11.25 μm x 11.25 μm, 22.5 μm x 22.5 μm, and 45 μm x 45 μm. Box density refers to the number of fibers within a specified area, while Distance to Nearest indicates the distance to the closest of a specified number of fibers. Alignment of Nearest and Box Alignment are calculated in relation to the nearest of a specified number of neighboring fibers and the alignment within a designated area, respectively; an alignment value of one indicates parallel alignment to the neighboring fibers, while a value of zero indicates that the fiber is perpendicular to the specified comparison set.

### Detection of ECM-Crosslinking Enzymes

Prior to immunohistological staining for lysyl oxidase (LOX) and procollagen-lysine,2-oxoglutarate 5-dioxygenase 1 (PLOD1), tissue sections were deparaffinized using xylene and rehydrated with decreasing concentrations of ethanol. Antigen retrieval was performed for 2 hours at 80°C in a citric acid solution (Vector Laboratories, Burlingame, CA). Staining for LOX was conducted using a rabbit polyclonal anti-LOX antibody (ab31238, Abcam, Cambridge, MA), while PLOD1 was detected using a rabbit polyclonal anti-PLOD1 antibody that recognizes the N-terminus (aa73-102) of human PLOD1 (ab171140, Abcam). Both colorimetric and fluorescent detection methods were used in order to yield images optimized for qualitative and quantitative analysis, respectively. For colorimetric detection, the Universal Vectastain Elite ABC system (Vector Laboratories) was used as the secondary antibody and combined with ImmPACT DAB Peroxidase (HRP) Substrate (Vector Laboratories). Tissues were then mounted using Aquamount and imaged on an Olympus IX51 inverted microscope. Fluorescence-based detection was conducted using an anti-rabbit biotinylated secondary antibody (Vector Laboratories) and Dylight-488 streptavidin tertiary antibody (Vector Laboratories). Tissue sections were mounted using Prolong Gold Antifade reagent and imaged. Quantification of LOX and PLOD1 expression was performed by capturing three fluorescent images per tissue layer using an Olympus IX51 inverted microscope. Using FIJI, the integrated intensity of three areas of equal dimensions per tissue layer per image was measured.

LOX and PLOD1 were also quantified in digested tissue samples using a modified dot blot method [32]. Tissue sections (10 μm thickness) were digested overnight at 55°C in a solution of >600 mAU/mL proteinase K (Qiagen, Hilden, Germany), diluted 1:8 in diH_2_O, followed by quantification of total protein content using a Micro BCA Assay kit (Thermo Fisher Scientific, Waltham, MA). Tissue digests were diluted to 5 μg/μL total protein, and 1 μL was loaded onto a PVDF membrane (Bio-Rad Laboratories, Hercules, CA) that had been soaked in methanol for one minute and rinsed in diH_2_O for 5 minutes. Collagen quantification was completed using a picrosirius red dot blot method described previously [32], utilizing rat tail collagen type I to create a standard curve from 0.1 to 0.8 μg/μL

LOX quantification was completed using the same tissue digests prepared for collagen quantification and diluted to 5 μg/μL total protein. A PVDF membrane (Bio-Rad) was prepared in the same manner as for collagen quantification, and a LOX standard curve (from 0.625 to 20 ng/μL) was created using a full-length human LOX protein (ab187448, Abcam). The membrane was loaded with 1 μL of each sample and standard. Standards and samples were fixed onto the membrane through incubation at 37°C for 5 minutes. Once fixed, the membrane was blocked in 5% non-fat, dry milk in 1x PBS for 1 hour at room temperature with shaking. Following blocking, the membrane was incubated overnight at 4°C with the same anti-LOX antibody (Abcam) used above for immunohistological staining, diluted 1:1000 in a solution of 1% dry milk and 0.1% Tween-20 in 1x PBS. The membrane was then washed 4 times in 0.01% Tween-20 in 1x PBS before incubating for 1 hour in a secondary antibody solution at room temperature with shaking. The secondary antibody solution consisted of an anti-rabbit IgG HRP conjugated antibody (#20320, Alpha Diagnostic Intl. Inc, San Antonio, TX) diluted 1:10000 in 1% dry milk, and 0.1% Tween-20 in 1x PBS. Following incubation with the secondary solution, the membrane was washed 4 times in 0.01% Tween-20 in 1x PBS. The membrane was then incubated in SuperSignal™ ELISA Pico Chemiluminescent Substrate (Thermo Fisher) before imaging. Membranes for both collagen and LOX quantification were imaged using a ChemiDoc™ MP (Bio-Rad) and analyzed using FIJI. Before measurement of signals, the Subtract Background command was used, and signals were measured using a circular selector of the same size for all standards and samples on membrane.

### *COL1A1* and *LOX* Gene Expression via qRT-PCR

Isolation of RNA from tissues sections for qRT-PCR was completed using the RNeasy FFPE kit (Qiagen), which is specifically tailored for the isolation of RNA from tissues that were previously formalin fixed and paraffin embedded (FFPE). RNA quantity and quality was determined via Nanodrop (Thermo Fisher Scientific) before creation of cDNA using a High-Capacity cDNA Reverse Transcription Kit (Applied Biosystems, Carlsbad CA). RT-PCR amplification was conducted using Taqman Gene Expression Assays (Applied Biosystems) for *Col1A1* (Hs00164004_m1) and *LOX* (Hs00180_m1). The ΔΔCt method was used to normalize to *GAPDH* (Hs02758991_g1) and determine relative expression of *Col1A1* and *LOX* genes compared to healthy samples.

### Statistical Analysis

Statistical analysis of SHG fiber characteristics was completed in Prism 6 (Graphpad Software, Inc., La Jolla CA) using a one-way ANOVA followed by a Tukey post hoc test. All other comparisons between two groups were completed using two-tailed, unpaired t-tests assuming unequal variance. Differences were considered to be significant when p<0.05. Each experiment was conducted using age-matched tissues from a total of 10 individuals (N = 5 healthy, N = 5 diseased) unless otherwise noted.

## Results

### Valve Classification

Histological measurement of leaflet cross-sections demonstrated an average leaflet thickness of 423.7 ± 163.0 μm for healthy specimens and 1866 ± 1032 μm for diseased specimens (p = 0.03434; Table 1). In all cases, the histological classification of healthy vs. diseased was consistent with the clinical evaluation of the valves. There was no statistically significant difference in the age of patients across the groups.

**Table 1:** Human valve specimen information.

Sex	Diseased	Age (yrs)	Mean Age (yrs) (p = 0.825)	Tissue Thickness (μm)	Mean Thickness (μm) (p = 0.034)
Female	N	50	64.2 ± 9.42	422.6 ± 88.60	423.7 ± 163.0
Female	N	61		206.4 ± 63.06	
Male	N	66		515.5 ± 137.6	
Male	N	69		341.1 ± 210.4	
Male	N	75		633.0 ± 127.6	
Female	Y	50	65.6 ± 9.92	872.9 ± 346.4	1866 ± 1032
Male	Y	62		3398 ± 1895	
Female	Y	69		1409 ± 526.3	
Male	Y	73		1229 ± 1090	
Male	Y	74		2422 ± 946.0	

### CAVD Is Accompanied by Layer-Specific Changes In Collagen Content

**Fig 1:**
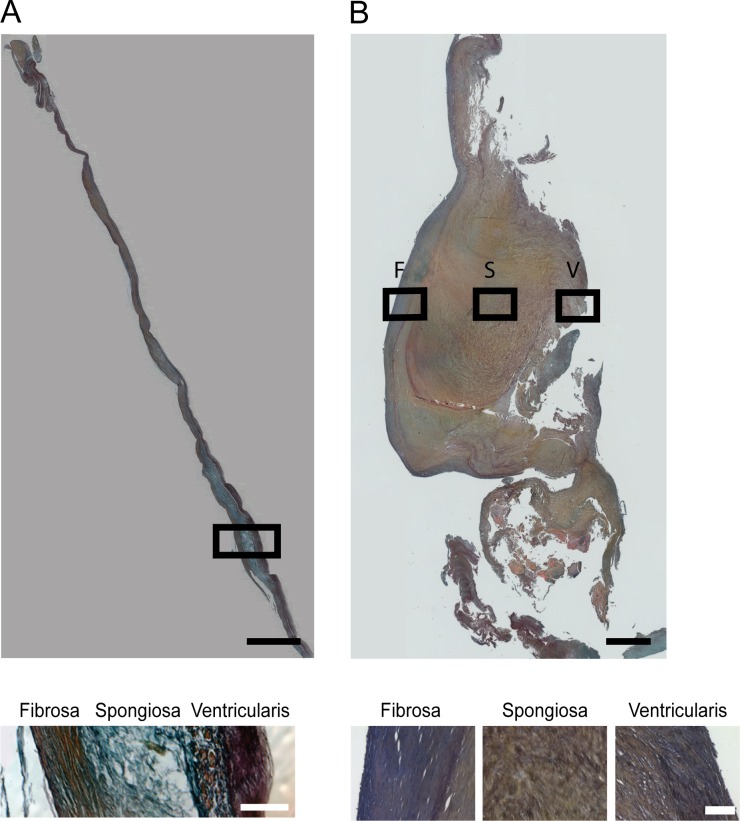
Representative images of human aortic valve leaflets stained with Movat’s Pentachrome. Top: Images of a complete leaflet are shown for a representative **(A)** healthy and **(B)** diseased valve. F = fibrosa, S = spongiosa, V = ventricularis. Scale bar = 100 μm. Bottom: Higher magnification images of the boxed area in the top images show the three leaflet layers in greater detail. Scale bar = 50 μm.

Movat’s pentachrome staining of healthy human aortic valve leaflets showed a defined trilaminar ECM architecture and uniform thickness (Fig 1A). Collagen (yellow) was prevalent in the fibrosa and ventricularis, while glycosaminoglycans (blue) comprised the spongiosa, and elastin (black/purple) was localized to the ventricularis (Fig 1B). This layered ECM structure was clearly disrupted in diseased leaflets (Fig 1A). Specifically, one of the most notable changes in the ECM of diseased valves was the enrichment of collagen (yellow) throughout the leaflet thickness (Fig 1B).

**Fig 2:**
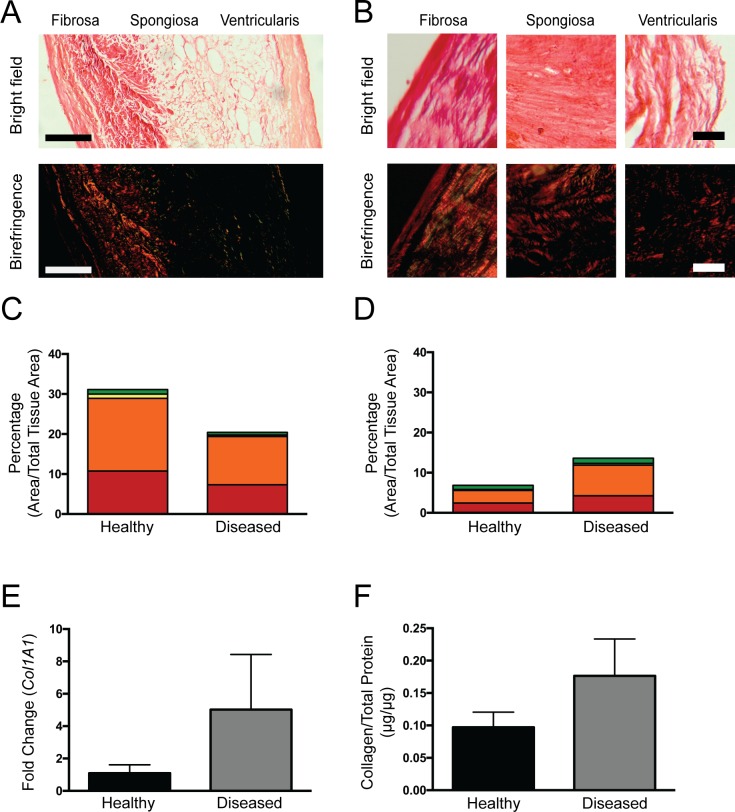
Visualization of collagen fibers via picrosirius red staining and quantification of collagen content. Picrosirius red staining of **(A)** healthy and **(B)** diseased leaflets was visualized using brightfield microscopy and polarized light. Birefringence hue and amount were quantified as a percent of total tissue area (N = 5, n = 3) in the **(C)** fibrosa and **(D)** spongiosa. Collagen production was also quantified via **(E)** qRT-PCR analysis of *COL1A1* gene expression (N = 4, n = 3) and **(F)** measurement of total collagen protein via dot blot (N = 5 healthy; N = 4 diseased, n = 3).

To provide a more targeted analysis of collagen content and initial characterization of fiber organization, leaflets were stained with picrosirius red and imaged under polarized light. The birefringent color of collagen fibers viewed in this manner is commonly related to fiber diameter (where increasing diameter corresponds to transition from green to yellow to orange to red color), although other fiber features such as packing density and alignment may influence color [33]. In healthy leaflets, the fibrosa was densely populated with collagen fibers showing mainly orange-red birefringence (Fig 2A). Sporadic, thin collagen fibers were observed in the spongiosa and exhibited a yellow-green color under polarization. In the healthy ventricularis, brightfield imaging of picrosirius red staining showed faint collagen presence, but few collagen fibers were detected under birefringence, likely due to fibers being orthogonal to the plane of imaging.

In diseased leaflets, the fibrosa was densely populated with collagen fibers of orange-red birefringence (Fig 2B), similar to the fibrosa of healthy leaflets. The spongiosa in diseased leaflets, however, was densely populated with orange-red birefringent collagen fibers, which was in contrast with the spongiosa of healthy leaflets where little to no birefringence was observed. The percent of the tissue area occupied by birefringent collagen fibers was decreased in the fibrosa of diseased valves relative to healthy valves (Fig 2C), but was increased in the spongiosa with disease (Fig 2D). However, because fiber orientation can also impact the presence of birefringence, the changes in collagen content inferred from the birefringence analysis may actually be due to a combination of changes in collagen content and collagen fiber orientation. Traditional tissue-level analyses did not show a statistically significant increase in overall leaflet collagen content (Fig 2E) or *COL1A1* mRNA (Fig 2F).

Although imaging of picrosirius red staining under polarized light enables improved visualization of collagen fibers relative to other histological methods, only limited quantitative information about their architecture could be obtained in our tissues with this approach. Quantification of the amount of each birefringent hue in the fibrosa and spongiosa revealed few differences between the healthy and diseased samples for each layer (S2 Fig). When comparing the spongiosa to the fibrosa, it appeared that the spongiosa was composed of more immature (i.e., thinner) fibers, as suggested by the presence of more green fibers. However, as noted above, the birefringence hue can change not only with fiber width, but also fiber density and alignment [33].

### CAVD Is Accompanied by Layer-Specific Changes In Collagen Architecture

**Fig 3:**
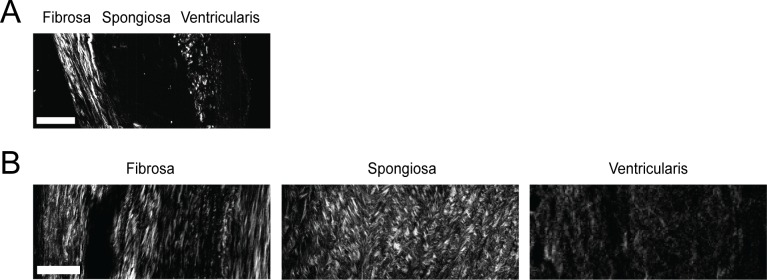
Visualization of collagen fiber microarchitecture using SHG microscopy. Representative images from **(A)** healthy and **(B)** diseased leaflets show microarchitectural changes in collagen in the fibrosa and spongiosa. Scale bar = 50 μm.

While picrosirius birefringence did reveal greater collagen fiber detail compared to Movat’s staining, SHG imaging was employed to achieve greater and more directly quantifiable detail about fiber characteristics. SHG imaging of the fibrosa in both healthy and diseased valves showed densely-packed, radially-aligned collagen fibers (Fig 3A and 3B; S1 Movie). The ventricularis in healthy valves contained collagen fibers oriented perpendicularly to those in the fibrosa, as viewed by fibers emanating outward from the viewing plane; the SHG signal from the ventricularis in diseased tissues was more diffuse, but the orthogonal imaging orientation of this layer precludes drawing conclusions about changes in fiber architecture. Finally, the most marked qualitative differences in collagen were seen in the spongiosa (S2 and S3 Movies). In contrast to the picrosirius red images, which showed some collagen fibers in the diseased spongiosa with limited distinction of individual fiber architecture, SHG imaging revealed an abundance of dense and disorganized collagen fibers in this layer, with clear distinction of individual fibers and their organization.

Quantitative analysis of SHG images yielded layer-specific data for characteristics of the individual collagen fibers. The ventricularis was omitted from this analysis for several reasons, including both significant logistical constraints and the consideration that this layer is generally not the site of valve calcification [34]. With respect to logistical constraints, accurate analysis of the fibers in the ventricularis would require *en face* leaflet sectioning to yield fibers laying within the imaging plane; because of the thinness of ventricularis, this approach would necessitate dedicated samples from a separate set of patients, with only one SHG section available per leaflet, and no ventricularis material available for complementary analyses (*e*.*g*., immunohistochemistry, biochemical assays, PCR). Importantly, such sectioning would also not be possible in diseased valves, which are highly amorphous (Fig 1B). Thus, our acquisition of fiber data focused on the fibrosa and spongiosa. In the fibrosa, development of CAVD was associated with surprisingly few changes to fiber characteristics; there was a significant decrease in fiber length and a change in fiber orientation in the diseased fibrosa, but fiber width, density, and alignment with neighboring fibers were not substantially changed (Table 2).

**Table 2:** Quantification of collagen fiber characteristics in the fibrosa and spongiosa of healthy vs. diseased aortic valve leaflets.

	FIBROSA		SPONGIOSA	
MEASUREMENT	HEALTHY AVERAGE (30,093 fibers)	DISEASED AVERAGE (36,052 fibers)	HEALTHY AVERAGE (12,922 fibers)	DISEASED AVERAGE (31,417 fibers)
**FIBER DIMENSIONS**				
Fiber Total Length (μm)	29 ± 2.3	25 ± 1.5^**F**^	22 ± 2.6^**F**^	22 ± 1.8
Fiber End-to-End Distance (μm)	26 ± 2.2	23 ± 1.6^**F**^	20 ± 2.4^**F**^	20 ± 1.8
Curvature	0.96 ± 0.0060	0.92 ± 0.0046	0.91 ± 0.0066^F^	0.91 ± 0.0057
Fiber Width (μm)	1.9 ± 0.13	1.9 ± 0.033	1.7 ± 0.025^**F**^	1.9 ± 0.089^**S**^
**FIBER DENSITY**				
Distance to Nearest 2	7.7 ± 0.23	7.4 ± 0.16	9.8 ± 0.55^**F**^	8.0 ± 0.77^**S**^
Distance to Nearest 4	10 ± 0.33	9.5 ± 0.20	13 ± 0.84^**F**^	10 ± 1.1^**S**^
Distance to Nearest 8	14 ± 0.51	13 ± 0.25	19 ± 1.8^**F**^	14 ± 1.6^**S**^
Distance to Nearest 16	19 ± 0.65	18 ± 0.35	29 ± 3.5^**F**^	20 ± 2.6^**S**^
Box Density 11.25 x 11.25 μm	1.7 ± 0.031	1.7 ± 0.043	1.5 ± 0.050^**F**^	1.7 ± 0.084^**S**^
Box Density 22.5 x 22.5 μm	4.4 ± 0.15	4.7 ± 0.17	3.4 ± 0.27^**F**^	4.3 ± 0.48^**S**^
Box Density 45 x 45 μm	15 ± 1.2	16 ± 0.65	9.9 ± 1.3^**F**^	14 ± 2.4^**S**^
**FIBER ALIGNMENT**				
Fiber Absolute Angle (°)	69 ± 15	89 ± 28^**F**^	73 ± 3.8	94 ± 26^**S**^
Alignment of Nearest 2	0.90 ± 0.020	0.86 ± 0.031	0.82 ± 0.059^**F**^	0.80 ± 0.047
Alignment of Nearest 4	0.86 ± 0.031	0.80 ± 0.045	0.73 ± 0.093^**F**^	0.71 ± 0.072
Alignment of Nearest 8	0.83 ± 0.037	0.77 ± 0.052	0.68 ± 0.12^**F**^	0.66 ± 0.092
Alignment of Nearest 16	0.82 ± 0.043	0.75 ± 0.057	0.64 ± 0.14^**F**^	0.63 ± 0.11
Box Alignment 11.25 x 11.25 μm	0.94 ± 0.014	0.91 ± 0.023	0.91 ± 0.027	0.88 ± 0.020
Box Alignment 22.5 x 22.5 μm	0.86 ± 0.030	0.80 ± 0.045	0.78 ± 0.072	0.74 ± 0.057
Box Alignment 45 x 45 μm	0.82 ± 0.043	0.76 ± 0.057	0.69 ± 0.11	0.64 ± 0.093

**F**: p<0.05 vs. healthy fibrosa

**S**: p<0.05 vs. healthy spongiosa

Meanwhile, the nature of the changes in collagen fiber architecture that occurred in the diseased spongiosa had minimal overlap with the type of changes observed in the diseased fibrosa. For example, CAVD was associated with a decrease in fiber length and no change in width for collagen in the fibrosa, but collagen fibers in the spongiosa exhibited no change in length and a significant increase in width in diseased leaflets (Table 2). Consistent with qualitative examination of SHG images and S2 and S3 Movies, quantitative fiber analysis confirmed a substantial increase in fiber number and density in the spongiosa of diseased valves relative to healthy conditions.

The boundary between the fibrosa and spongiosa in healthy leaflets is usually marked by a sudden decrease in collagen staining, which is consistent with our quantification of collagen fibers across these layers. Specifically, Table 2 shows significant differences between the healthy fibrosa and spongiosa for almost every collagen fiber parameter examined: total number, length, width, density, and alignment. However, in diseased leaflets, the abundance of collagen in the spongiosa appeared to blur this boundary, and the fibrosa and spongiosa became difficult to distinguish from each other both qualitatively and quantitatively. These two layers went from differing in almost all measurement areas in a healthy valve to no significant differences in any fiber feature in the diseased valve (Table 2). This increase in layer similarity in diseased valves appeared to be driven by fibers in the spongiosa becoming more similar to those in the fibrosa. For example, although fibers in the healthy spongiosa were much thinner than those in the healthy fibrosa (as observed in Fig 3 and Table 2), the thickening of fibers solely in the diseased spongiosa ultimately yielded an average fiber width that did not differ from that found in the corresponding fibrosa.

### Increased LOX Expression in Diseased Fibrosa

**Fig 4:**
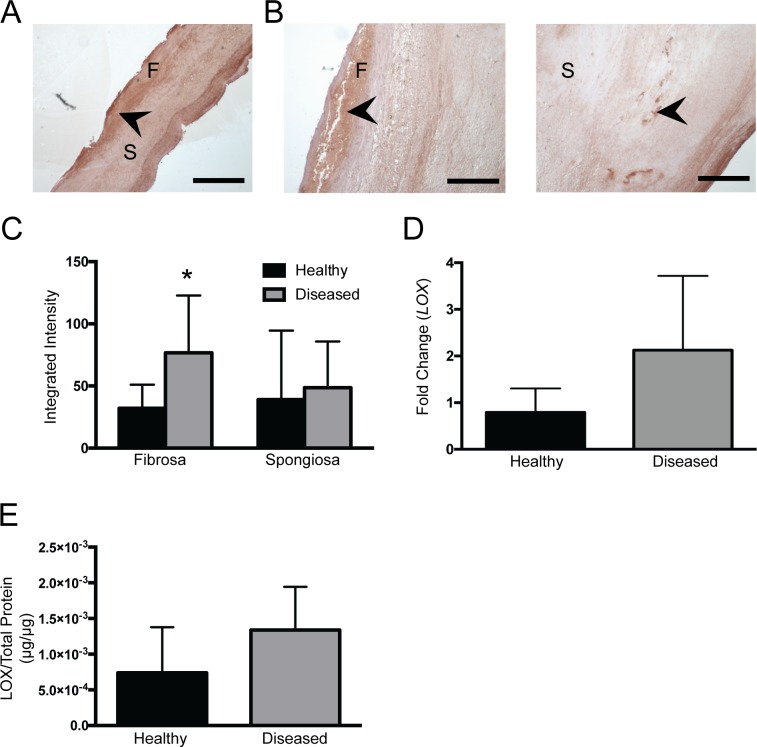
Immunohistochemical detection and quantification of lysyl oxidase (LOX) expression. Immunohistochemical staining of **(A)** healthy and **(B)** diseased leaflets shows distribution of LOX production throughout leaflet, with some areas of localized high-intensity staining (indicated by arrows). F = fibrosa, S = spongiosa. N = 5. Scale bar = 0.50 mm. **(C)** Layer-specific quantification of LOX staining, indicating significantly greater amounts of LOX in the diseased fibrosa relative to the healthy condition (p = 0.001728). N = 5, n = 3. Total LOX expression across the entire leaflet was also quantified via **(D)** qRT-PCR for LOX (N = 5, n = 3) and **(E)** measurement of LOX protein via dot blot (N = 5, n = 3).

We next investigated whether CAVD was associated with an increase in the production of enzymes involved in post-translational modification of collagen, specifically PLOD and LOX, as these molecules are capable of effecting changes in collagen fibrillar structure [35, 36]. PLOD is responsible for converting triple helical lysyl residues into hydroxylysine groups; this modification is a required step in the formation of fiber crosslinks and collagen maturation [36]. Immunohistochemical staining for PLOD1 showed relatively uniform presence of this enzyme across all leaflet layers, with no quantitative differences between healthy and diseased leaflets (S3 Fig). We also measured the presence and expression of LOX, which catalyzes the formation of highly reactive aldehydes from lysyl residues and comprises the rate-limiting step in forming stable collagen fibrils [35]. In both healthy and diseased tissues, LOX was prevalent in the fibrosa and ventricularis, with fainter expression throughout the spongiosa (Fig 4A and 4B). However, the spongiosa of diseased leaflets also contained localized areas of intense LOX staining, which were not seen in healthy valves. The expression of LOX was quantified via fluorescent staining, as shown in Fig 4C. While LOX expression was not significantly increased in the diseased spongiosa, there was a significant increase in LOX expression in the diseased fibrosa. Quantification of *LOX* mRNA and protein across the entire leaflet structure did not yield any statistically significant differences between healthy and diseased tissues (Fig 4D and 4E).

## Discussion

Previous studies of valves with CAVD have noted an increase in collagen disorganization using traditional histological analysis techniques [6, 37]. However, the nature of this disorganization has not been clearly described, nor have its causes been elucidated. Although collagen fiber orientation in healthy human aortic valves has previously been characterized [14], the current work is the first to quantify collagen fiber characteristics in human valves with CAVD. Additionally, this work examined fiber architecture in healthy valves from elderly individuals (>65 years old), which has not previously been done, but is particularly important in the context of CAVD, where advanced age is the primary risk factor. Through the application of advanced imaging modalities, we were able to conduct a quantitative, layer-specific characterization of individual collagen fibers in these healthy and diseased aortic valves, demonstrating differences in the distribution, architecture, and post-translational modification of collagen in CAVD. Together, these findings reveal a more complex picture of collagen enrichment and arrangement than has previously been described using traditional analysis methods, both with respect to the level of architectural quantification achieved using SHG, as well as the layer-specific quantification of collagen characteristics. The results from this analysis also motivated the quantification of collagen crosslinking enzymes, which have not been previously examined in healthy or diseased human aortic valves.

The combination of layer-specific analysis and fibrillar quantification revealed surprisingly few changes to the fibrosa during disease; fibers in the diseased fibrosa were significantly shorter than those in the healthy fibrosa, but otherwise had similar overall fiber number, width, density, and alignment. It should be noted that measurements for fiber length may be affected by fibers exiting the plane of imaging, and a change in fiber length could also be due to a change in fiber alignment out of the circumferential direction. However, fiber analyses of single-plane images from z-stacks yielded statistically similar length values as analyses of multiple merged images from z-stacks, which suggests that the changes in fiber length are not likely to be artifacts related to fiber clipping. In contrast to the fibrosa, the spongiosa underwent substantial changes to its collagen fiber structure with disease. There was a >2-fold increase in total number of collagen fibers in the diseased spongiosa, where the fibers maintained constant length, but became significantly wider and denser. These types of structural changes are important because they have the ability to alter both cellular behaviors and valve mechanical properties [38–40]. As noted earlier, features such as fiber width, density, and alignment can regulate cell polarity, proliferation, migration, ECM production, and differentiation [20, 38]. Fiber alignment may also enjoy a reciprocal relationship with VIC orientation, as an investigation of porcine VICs in 3D collagen gels cultured under anisotropic cyclic strain found that increased VIC orientation can precede increased collagen fiber alignment [41]. VIC orientation may be an interesting parameter to evaluate in future studies of human valve disease, as it was found to strongly influence VIC phenotype *in vitro* [41].

Performance of our analysis in a layer-specific manner was also important in order to characterize the spatial heterogeneity in valve ECM rearrangement that was not evident in full-tissue analyses. Considering both the collagen quantification and LOX results together, it is possible that the changes in collagen architecture in the fibrosa and spongiosa are proceeding via different mechanisms. Specifically, because the diseased fibrosa exhibits decreased fiber length, little change in fiber number or density, and an increase in LOX, we speculate that collagen degradation is outpacing collagen biosynthesis in the diseased fibrosa, while crosslinking of existing fibers is increased. In contrast, the increase in fiber number and density in the spongiosa, without any change in LOX, could be an indication that the spongiosa is in a phase of elevated collagen production [42, 43]. These potential explanations are consistent with the picrosirius red birefringence analysis, which showed an abundance of mature collagen fibers in the fibrosa, and a greater proportion of immature fibers in the spongiosa (S2 Fig).

Relating this work to existing literature on the ECM in sclerotic and stenotic valves, collagen “disorganization” is a common hallmark of CAVD [37], and our analysis indicates that the collagen appears disorganized primarily due to it being strongly enriched in the spongiosa, rather than large-scale changes in collagen orientation or alignment. It is also useful to note that the general fibrillar arrangement within our healthy valve cohort appears qualitatively similar to that described for adult porcine valves [17, 18]. With respect to collagen remodeling, significantly increased production of both matrix metalloproteinases (MMPs) and their inhibitors (TIMPs) in human valves with CAVD has previously been described [44–46]; while some have found an imbalance favoring MMP production [44], others have hypothesized a more nuanced situation where the production of these molecules depends upon the local environment within the valve [46]. Our findings of differential collagen remodeling in different layers of the leaflet are in agreement with this hypothesis. Expression of collagen crosslinking enzymes has not previously been evaluated in human aortic valves, but an analysis of healthy porcine valves found more LOX in the spongiosa compared to the fibrosa of young, healthy valves, and an increase in the overall presence of LOX with advanced age [47]. Our findings differ in that we did not observe differences in the amount of LOX in the fibrosa vs. spongiosa in healthy human valves; this could be due to a difference in species, difference in age relative to the swine, or just greater heterogeneity amongst humans. The layer-by-layer approach to quantifying ECM architecture that was employed in this study may also be applied to further our understanding the layer-specific nature of valve calcification in human valves, where calcific deposits occur preferentially in the fibrosa [34]. Although many features likely contribute to this layer-specific nature of calcification, collagen architecture has not previously considered as a factor that may influence this process; other studies of bone mineralization indicate that fiber organization can indeed regulate calcification [48].

Alterations in collagen fiber organization are also likely to impact the mechanical properties of the valve. The opposing alignment of collagen fibers in the fibrosa and ventricularis in healthy aortic heart valves is critical for maintaining appropriate opening and closure of the valve [2]. Significant changes in the orientation angle of fibers in both the diseased fibrosa and spongiosa, as well as the overall increase in collagen fibers in the spongiosa, may indicate that mechanical loads are being supported outside of the planes of mechanical stress that would be typical for a healthy aortic valve leaflet. This may be especially true for the spongiosa, which normally works to minimize friction between the stress-bearing fibrosa and ventricularis during healthy aortic valve function. An increase in collagen fiber density is likely to impede the ability of the spongiosa to insulate the mechanical properties of the fibrosa from the ventricularis and vice versa. Disruptions to the mechanical properties of the valve may not only influence its physical functionality, but also cellular behavior. For example, *in vitro* investigations have shown that increased substrate stiffness causes an increase in the expression of disease markers such as αSMA and apoptosis by VICs [49].

Finally, the use of high-resolution SHG in this work was found to enable a level of collagen fiber characterization not possible with histological approaches. The application of new approaches and complementary tools to characterize ECM organization is important for understanding the etiology of fibrotic diseases such as CAVD. Because SHG does not require tissue fixation or staining, its use could advance current efforts to engineer fibrotic environments *in vitro* [50]. The combination of fibrosis tissue engineering with SHG may offer the ability to gain insight into fibrogenic processes through the non-invasive visualization of ECM dynamics in living cultures [51]. Recent work has also shown that SHG imaging of collagen alterations may be combined with machine learning algorithms to characterize the extent of fibrosis in clinical samples, as well as provide automated, high-throughput diagnoses of fibrotic disease [52–56]

## Conclusions

The ECM, and specifically, collagen, is known to regulate cell behavior not only through its primary amino acid sequence, but also through its architecture and organization. Prior to the current work, the nano/micro-scale architecture of collagen had not been quantified in human valves with CAVD. The responsiveness of many other cell types to changes in nano/micro-topographies and the changes in collagen fiber structure described herein motivate future investigations into whether these changes in ECM architecture are capable of influencing VIC function and/or calcification. The implications for this work range from gaining insight into the biological and mechanical events in valve pathology to informing the creation of scaffold environments for tissue engineering of valves.

## Supporting Information

S1 Fig Representative images of a diseased leaflet (left) and a healthy leaflet (right) used in this study. Arrows indicate macroscopically evident calcified nodules.
(PDF)

S2 Fig Distribution of birefringent hues in the fibrosa (A) and spongiosa (B), expressed as a percent of total birefringence.
(PDF)

S3 Fig Immunohistochemical detection of PLOD1 in healthy and diseased aortic valve leaflets. Arrows indicate localized areas of positive PLOD1 staining. N = 5.
(PDF)

S1 Movie 3D rendering of SHG images of collagen architecture in the fibrosa and spongiosa of a healthy valve. (MOV)

S2 Movie 3D rendering of SHG images of collagen architecture in the spongiosa of a healthy valve. (MOV)

S3 Movie 3D rendering of SHG images of collagen architecture in the spongiosa of a diseased valve. (MOV)
