# Supplementary material for: Calcific Aortic Valve Disease Is Associated with Layer-Specific Alterations in Collagen Architecture
Source: PLoS One. 2016 Sep 29;11(9):e0163858. doi: 10.1371/journal.pone.0163858 (PMC5042542; doi:10.1371/journal.pone.0163858)
Supplement: S1 Fig — Arrows indicate macroscopically evident calcified nodules. (PDF) [file pone.0163858.s001.pdf]

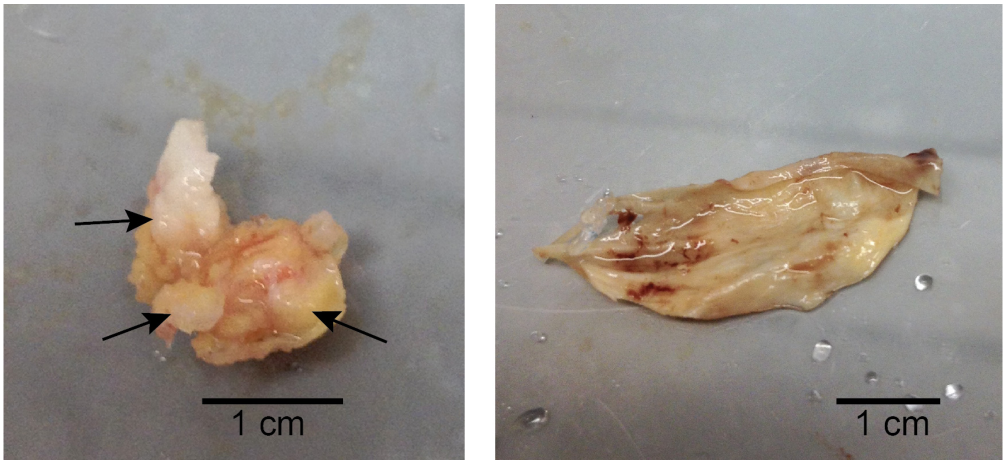

Figure S1. Representative images of a diseased leaflet (left) and a healthy leaflet (right) used in this study. Arrows indicate macroscopically evident calcified nodules.
