# Supplementary material for: Calcific Aortic Valve Disease Is Associated with Layer-Specific Alterations in Collagen Architecture
Source: PLoS One. 2016 Sep 29;11(9):e0163858. doi: 10.1371/journal.pone.0163858 (PMC5042542; doi:10.1371/journal.pone.0163858)
Supplement: S2 Fig — Distribution of birefringent hues in the fibrosa (A) and spongiosa (B), expressed as a percent of total birefringence. (PDF) [file pone.0163858.s002.pdf]

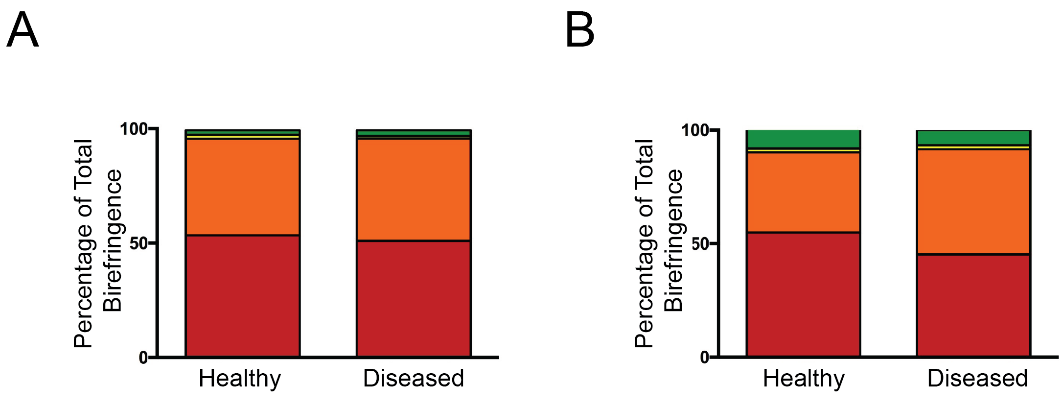

Figure S2. Distribution of birefringent hues in the fibrosa (A) and spongiosa (B), expressed as a percent of total birefringence.
