# Supplementary material for: Calcific Aortic Valve Disease Is Associated with Layer-Specific Alterations in Collagen Architecture
Source: PLoS One. 2016 Sep 29;11(9):e0163858. doi: 10.1371/journal.pone.0163858 (PMC5042542; doi:10.1371/journal.pone.0163858)
Supplement: S3 Fig — Arrows indicate localized areas of positive PLOD1 staining. N = 5. (PDF) [file pone.0163858.s003.pdf]

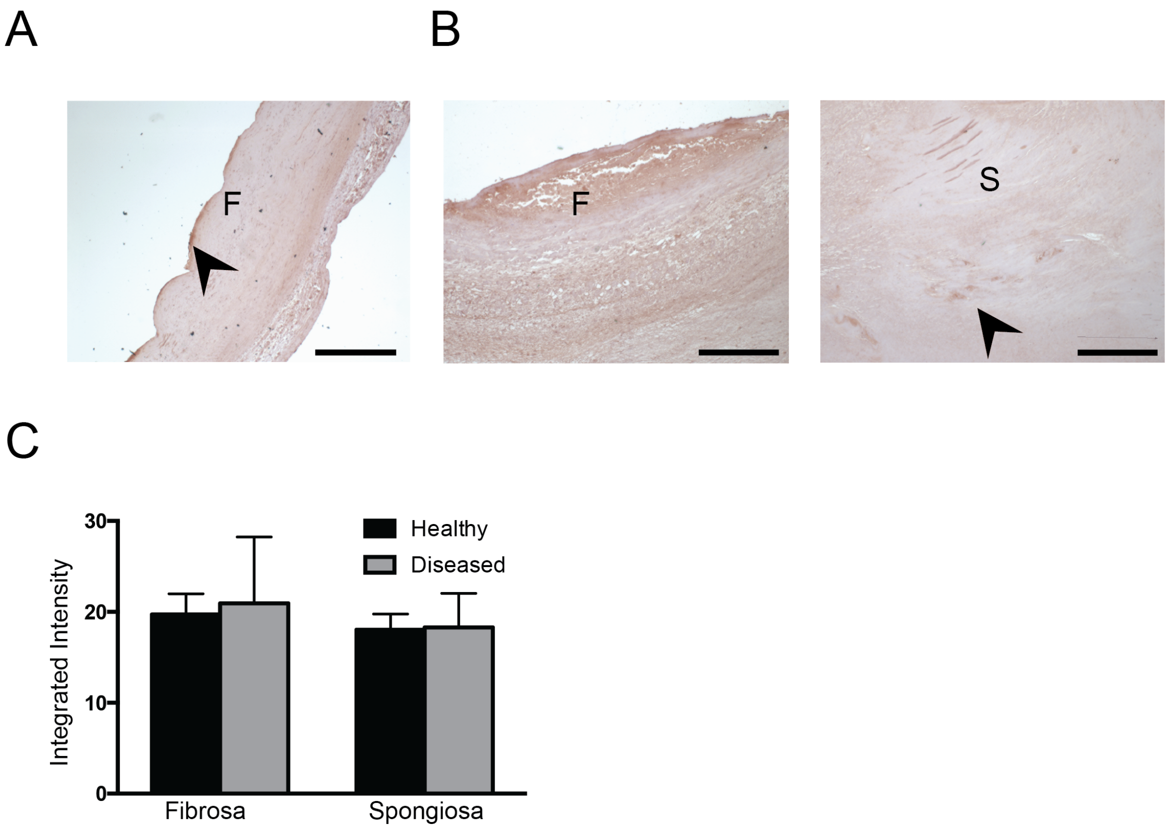

Figure S3. Immunohistochemical detection of PLOD1 in healthy and diseased aortic valve leaflets. Arrows indicate localized areas of positive PLOD1 staining. N=5.
